# Estimating the future burden of cardiovascular disease and the value of lipid and blood pressure control therapies in China

**DOI:** 10.1186/s12913-016-1420-8

**Published:** 2016-05-10

**Authors:** Warren Stevens, Desi Peneva, Jim Z. Li, Larry Z. Liu, Gordon Liu, Runlin Gao, Darius N. Lakdawalla

**Affiliations:** Precision Health Economics, Oakland, CA USA; Precision Health Economics, Los Angeles, CA USA; Pfizer Inc, San Diego, CA USA; Weill Medical College of Cornell University, New York City, NY USA; Peking University National School of Development, Beijing, China; Fu Wai Hospital, National Center for Cardiovascular Diseases, Chinese Academy of Medical, Sciences and Peking Union Medical College, Beijing, China; University of Southern California, Los Angeles, CA USA

**Keywords:** Cardiovascular disease, Social value, Epidemiology, Lipids, Blood pressure, China

## Abstract

**Background:**

Lifestyle and dietary changes reflect an ongoing epidemiological transition in China, with cardiovascular disease (CVD) playing an ever-increasing role in China’s disease burden. This study assessed the burden of CVD and the potential value of lipid and blood pressure control strategies in China.

**Methods:**

We estimated the likely burden of CVD between 2016 and 2030 and how expanded use of lipid lowering and blood pressure control medication would impact that burden in the next 15 years. Accounting for the costs of drug use, we assessed the net social value of a policy that expands the utilization of lipid and blood pressure lowering therapies in China.

**Results:**

Rises in prevalence of CVD risk and population aging would likely increase the incidence of acute myocardial infarctions (AMIs) by 75 million and strokes by 118 million, while the number of CVD deaths would rise by 39 million in total between 2016 and 2030. Universal treatment of hypertension and dyslipidemia patients with lipid and blood pressure lowering therapies could avert between 10 and 20 million AMIs, between 8 and 30 million strokes, and between 3 and 10 million CVD deaths during the 2016–2030 period, producing a positive social value net of health care costs as high as $932 billion.

**Conclusions:**

In light of its aging population and epidemiological transition, China faces near-certain increases in CVD morbidity and mortality. Preventative measures such as effective lipid and blood pressure management may reduce CVD burden substantially and provide large social value. While the Chinese government is implementing more systematic approaches to health care delivery, prevention of CVD should be high on the agenda.

**Electronic supplementary material:**

The online version of this article (doi:10.1186/s12913-016-1420-8) contains supplementary material, which is available to authorized users.

## Background

The consequences of population aging in China are likely to be exacerbated by the rising prevalence of chronic, non-communicable diseases. The bulk of disease burden in China has shifted away from infectious diseases and reproductive complications in the last two decades, [1] with the prevalence of non-communicable diseases rising rapidly and now accounting for over 80 % of the overall disease burden [2]. Cardiovascular disease (CVD) plays the leading role among the set of non-communicable diseases, with stroke, ischemic heart disease, and chronic obstructive pulmonary disease as the leading causes of death in China in 2010 [1]. About 230 million patients, or approximately one in five adults in China, suffer from CVD [3]. The *Global Burden of Disease* reported that stroke was the leading cause of death in China in 2010, with coronary heart disease (CHD) close behind and gaining ground [4, 5]. In general, the CVD burden in China is expected to rise as the Chinese population becomes more sedentary and diets become more geared towards red meat, sugars, and saturated fats [3].

Evidence of this epidemiological transition has already been highlighted in China, [6] most recently with the aid of the *Global Burden of Disease* series [1]. These studies suggest that the proportion of total deaths attributable to CVD is rising at approximately 2 % annually, more quickly in urban areas than in rural areas [6]. The studies also highlight that although age is a strong predictor of CHD risk, the total increase in mortality rates is greater than the increase in mortality rates attributable solely to aging. The age-standardized CHD mortality rate has been rising at 1 % annually in the last decade, which accounts for about half of the total increase in mortality rates.

Although CVD factors in China are approaching those in Western nations, the use of primary and secondary prevention technologies has lagged behind that of the US and Europe, which have seen declines in CVD deaths partly due to expanded use of evidence-based CVD therapies [7]. In addition, the benefits of better CVD screening and prevention have not been realized in China. For example, around 30 % of hypertension in the Chinese population remains undiagnosed, [8] which is almost four times the corresponding rate in the US [9]. However, this suggests that Chinese health policymakers are presented with a major opportunity to prevent CVD by using one or more available prevention approaches. This was recognized by the Chinese government in a recent policy report suggesting that impending health policy reforms should highlight a shift from a health system that has traditionally been treatment-based towards one which has a greater emphasis on primary and secondary prevention [10].

A number of health care technologies and public health interventions have been shown to be successful in slowing the rise in the burden of CVD in industrialized countries. Systematic reviews demonstrate that prevention strategies for CHD and heart failure reduce hospital admissions; [11] enhance quality of life; [11, 12] improve health outcomes; [12–15] and reduce healthcare costs [12]. While some reviews have reported uncertainty about survival improvement; [11] recurrent cardiovascular incidents; [11] and cost effectiveness; [11, 12] others have seen statistically significant improvement in mortality; [16] cardiac events; [16] and gains in cost per quality-adjusted life years [15].

In addition to the more widely accepted use of blood pressure medication in China, statins have been shown to have a major impact on reducing CVD burden in many other countries [17]. The efficacy of statins in lowering the risk of cardiovascular events is well recognized around the world [18–21]. The EVANS study in France found that if statin-treated patients discontinued use, 4992 major cardiovascular events and 1159 deaths would occur in the first year after discontinuation [20]. The cost effectiveness of statins has varied across studies depending on the drug price and the patient’s level of risk. In general, patients who have higher baseline cardiovascular risk recoup comparably larger health and economic benefits [22–25]. Moreover, as the price of the drug falls, the cost-effectiveness of statins improves across all risk groups [25, 26]. Despite this, the current use of statins in low-income countries remains low. According to the Prospective Urban Rural Epidemiological study, the level of statin use was just 1.7 % in China, much lower than levels in the US and Europe (50–60 %) [27].

The Chinese government is currently working towards a more systematic approach to managing hypertension and diabetes. However, healthcare professionals and administrators only recently began to pay more attention to hyperlipidemia. Epidemiological transition theory [4] and recent evidence of growth in cholesterol levels [28] suggest that this is about to change. The current stage of evolution of the Chinese healthcare system presents a unique opportunity to intervene, due to its near universal health insurance coverage, the introduction of the New Rural Cooperative Medical System in 2003, and other schemes for employees of private and state-owned enterprises. Moreover, many insurance schemes in China have annual caps on inpatient and outpatient benefits, with coinsurance rates for inpatient care of 60 % [29]. The limits on inpatient coverage place even greater importance on the coverage of primary and secondary prevention. Measures aimed at improving lipid management and CVD prevention in China should play a significant role in this regard. While some studies have examined the burden of CVD in China with an aging population, there is a dearth of literature comprehensively accounting for the impact of China’s epidemiological transition and estimating the potential value of measures to mitigate the rise in mortality and morbidity from non-communicable disease. This study assessed the burden of CVD and the potential value of lipid and blood pressure control strategies in China.

## Methods

### Data sources

We obtained baseline data on age, gender, and prevalence of risk factors from the 2009 China Health and Nutrition Survey (CHNS), [28] a longitudinal health survey undertaken every 2 years that contains information on the prevalence of CVD risk factors such as hypertension, obesity, smoking, and diabetes, as well as levels of both high-density and low-density lipoprotein cholesterol (LDL-C) [28]. We used the 2009 wave, because it was the only year available with full biomarker data (sampling weights were not available in Popkin et al. 2009). It included almost 30,000 individuals in 15 provinces and municipal cities in China (see Additional file 1 for a more detailed description of study data). Since the CHNS data only include historical self-reported recall of CVD events, we estimated the incidence and mortality risk of both acute myocardial infarction (AMI) and stroke (hemorrhagic and ischemic) from the peer-reviewed literature (see Additional files 1, 2, and 3 for more detail). Longitudinal population trends were modeled using the United Nations Population Division predictions [30]. Preventative intervention effects were modelled using the World Health Organization (WHO) region-specific bivariate risk model [31].

### Analytical approach

Our model has three components: burden estimation, trend extrapolation, and an aversion estimation based on intervention.

#### Burden model

CVD burden was divided into five age strata (35–45, 45–55, 55–65, 65–75, and 75+ where lower limits are inclusive) separately for males and females. We further subdivided each of these age-sex subgroups into risk categories based on the number of metabolic syndrome conditions (0, 1 or 2, and 3+) from which they suffered (see Additional file 1 for metabolic syndrome definition cut-offs). With different risk profiles stroke events were divided into hemorrhagic stroke and ischemic stroke. Baseline AMI and stroke mortality and incidence were estimated from recent literature. Relative risks of CVD events for each of our strata age * risk group * gender were estimated using the methodology described in Additional file 1. The absolute burden was then calculated by multiplying the relevant CVD event rates by the age and gender stratified UN population data [30] and distributing between risk groups using the estimated relative risks.

#### Trend extrapolation

We project forward the incidence of CVD events in two ways. First, we model the trend in CVD events that would result from changes in the age-sex profile of the Chinese population, holding risk factors, relative risks, and all other factors constant. Second, we include projections of how underlying risk factors are likely to evolve in the future.

**Fig. 1:**
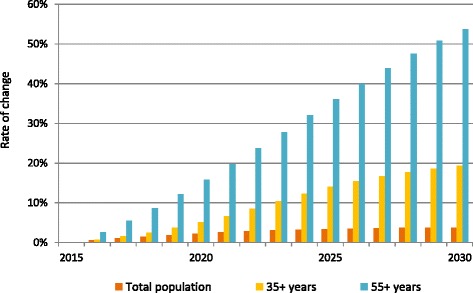
Projected rate of change in Chinese population (2016–2030). Notes: Data are based on the United Nations Population Division 2012 Project Population Forecasts POP/DB/WPP/Rev.2012/POP/F15-1

To project changes in the demographic profile over the next 15 years, we used United Nations Population Division projections for the Chinese population (Fig. 1), [30] stratified by age as shown in Additional file 1: Figure S3.1. Under this model the risk factor prevalence within each demographic subgroup stays constant.

Our second approach layers trends in risk factors on top of the projected demographic changes. To estimate the future prevalence of key risk factors, we utilized the theory of epidemiological transition to estimate linear regression models (using a robust regression algorithm based on multivariate weighted least squares solution with a bisquare weight function) for each risk factor prevalence as a function of gross national income per capita [32]. The models were estimated using data from 2000–2008 for 18 Southeast Asian countries (see Additional file 1: Figures S3.2 and S3.3 for risk factor trends) [33, 34]. This approach yields risk factor prevalence trends for each risk factor, which was converted to the model risk groups based on the number of subjects with 0, 1–2, or 3+ metabolic syndrome conditions. The risk factor prevalences are highly correlated, and the correlation structure was estimated from the CHNS biomarker data and assumed to remain constant over time. With the prevalence of each risk factor and their correlation known, subejcts’ dichotomous disease status for each risk factor were simulated from a multivariate binomial distribution. Using this simulated data, we then estimated the expected number of individuals in each of the model risk categories of 0, 1–2, or 3+ conditions. A detailed description of this process is provided in Additional file 1.

#### Intervention model

CHNS biomarker data were used to estimate the joint bivariate distribution of total cholesterol (TC) and systolic blood pressure (SBP) within each age * condition category for diabetic/non-diabetic and smoking/non-smoking subjects. The CVD event burden for each TC * SBP cell of the bivariate distribution within each strata was then estimated using the WHO CVD risk model [31]. For each intervention a prescription region is defined within the bivariate TC (LDL-C) * SBP distribution. A detailed derivation of this process is described in Additional file 1.

The intervention effects were defined by two components: the estimated average reduction in TC (LDL-C) and/or SBP magnitude, and the effect of that reduction on mortality and morbidity. Statins were assumed to reduce LDL-C by 38.7 mg/dL on average, with a 25 % reduction per 41.7 mg/dL (equivalent to 1 mmol/L) in AMI morbidity and mortality [35]. Ischemic stroke incidence was reduced by 20 % per 41.7 mg/dL, with no significant reduction (5 % level) in hemorrhagic stroke. There were no significant effects on stroke morbidity or mortality [36].

The reduction in SBP (delta_P) was estimated using the age-specific model, [37] where the reduction in SBP was assumed to be:

- ▪ delta_P1 = 9.1 + 0.1 * (SBP-154) for one drug
- ▪ delta_P2 = P1 + 9.1 + 0.1 * (SBP-P1-154) for two drugs
- ▪ delta_P = 0.5*(P1 + P2), where SBP is the population systolic blood pressure.

The accompanying risk in mortality and morbidity was calculated as F^(delta_P/20)^, where F takes the stroke (hemorrhagic and ischemic) and AMI specific values in Table 1 [37].

**Table 1:** Parameters for antihypertensive risk reduction model

Age	39.5	44.5	54.5	64.5	74.5	84.5	87.5
F_stroke_	0.36	0.36	0.38	0.43	0.50	0.67	0.67
F_AMI_	0.49	0.49	0.50	0.54	0.60	0.70	0.70

Data are based on Law et al. (2009)

The two-dimensional risk distribution allows a simultaneous statin and antihypertensive prescription model. Assuming for a particular population that P_LDL-C_ and P_SBP_ were the prescription cut-off points, we modeled the provision of statins and/or hypertensives using the following rules:

- ▪ Population A: LDL-C > P_LDL-C_ and SBP < P_SBP_ (statins)
- ▪ Population B: LDL-C < P_LDL-C_ and SBP > P_SBP_ (antihypertensive)
- ▪ Population C: LDL-C > P_LDL-C_ and SBP > P_SBP_ (both)

To avoid double counting of CVD events and to account for possible antagonism across therapies, we exploit research on polypill prescriptions [38] that estimates the total events averted as:

$$ \begin{array}{l} Events\  averted\  in\  Population\ A + Events\  averted\  in\  Population\ B + Events\  averted\  from\  antihypertensive\ \\ {} in tervention\  in\  Population\ C + 69.8\%\ *\  Events\  averted\  from\  statin\  in tervention\  in\  Population\ C.\end{array} $$

A flow diagram describing the three steps of burden, trend extrapolation, and intervention/aversion is provided in Additional file 1.

#### Translation to net social value

In order to estimate the potential cost and value to society as a whole, we estimated the cost of treatment for eligible patients over the time period of the study, and the cost savings from reduced CVD events. The mean annual cost estimate per person-treatment for hyperlipidemia or hypertension was taken from the literature. To estimate the cost of statin use for each patient over the 15-year period, we took initial estimates for an annual supply of statins of $291 (1819 RMB) from Zeng et al. (2013), weighted by originator drug and generic volumes data from the same study [39]. We multiplied this full year cost by the mean adherence level of 75 % from statin effectiveness studies, [40] to obtain an average prescription cost of $218 per person-year. Studies have shown a downward trend of just under 3 % in prices for both originator and generic statins in China recently (2004–2013) as utilization rises, [41] which we applied to future years (see Additional file 1 for more details).

A number of medications are used to treat blood pressure including diuretics, beta-blockers, alpha-blockers, angiotensin receptor blockers, calcium channel blockers, and angiotensin-converting enzyme inhibitors, and on average, the number of drugs taken by a hypertensive patient is 1.6 [42]. We took estimates of the annual cost of pharmaceutical treatment of hypertension from Le et al. (2012) with a mean of $130 per year for all drugs for each hypertensive patient in 2010 [43].

Estimates of the cost of events were also derived from the literature. Unlike new pharmaceuticals, these costs tend to rise over time, so we took recent estimates of AMI and stroke costs and applied 4.6 % annual estimate of the health care price index over the last 5 years for China [44]. We used this figure over the 15 years of the model. For AMI the mean cost was $6623 (RMB 46,365) in 2012, [40] and for stroke the mean cost was $1602 (RMB 11,216) in 2010 [45].

In order to estimate the potential social value of the averted CVD deaths, we considered several estimates of the value of life from value of statistical life (VSL) studies. Recent reviews on estimating the value of life have suggested estimates in the range of $500,000 to $2 million, although most of these studies have been undertaken in Europe and North America [46]. We found three relatively recent VSL estimates from studies undertaken in a Chinese population [47–49]. These studies estimated a mean VSL for various Chinese populations of 15–20 times gross domestic product (GDP) per capita. Our life tables suggested a value of a life year ranging from one to two times GDP per capita. Another approach developed by the WHO Macroeconomic Commission on Health meeting in 2000 suggests a value ranging from one to three times GDP per capita. Therefore, for our model, we used two times per capita GDP, which lies in the midpoint of the full range of values described by these studies. This results in an estimate of $13,408 per life year saved—twice the current GDP per capita of $6704. (Additional file 1: Appendices 8.1 and 8.2 provide sensitivity analysis of social value estimates based on varying value of a life year at 1 and 3 times GDP per capita.) We then used the age at death generated by the disease burden model and the most recent life tables for the Chinese population to generate life years lost at each age of death. Additional file 1 shows the methodology and the life tables we used to estimate life years saved from premature death averted.

## Results

### Future burden of CVD in China

The results of the model were consistent with a number of other projections of heart disease in China and Southeast Asia in that the burden of CVD is on the rise, and that primary and secondary prevention are likely to be key priorities for health policy in the near future [1, 50]. The model predicted that the number of AMIs is expected to rise from 4 million in 2016 to 6.1 million in 2030; strokes will rise from 6.1 million in 2016 to 9.8 million in 2030, and total deaths from CVD will rise from 2 million to 3.3 million per year during the same time period, keeping all other factors constant. Table 2 shows the total predicted number of CVD events and deaths in China over the 2016–2030 period by age and gender.

**Table 2:** Estimates of number of AMIs, strokes and CVD deaths by gender and age (2016–2030)

	35–45	45–55	55–65	65–75	Over 75
AMI incidence (2016–2030)					
Male	2,733,082	5,506,290	9,434,189	11,994,178	9,030,523
Female	2,111,375	4,634,030	9,143,257	11,232,330	9,199,603
Total	4,844,457	10,140,320	18,577,446	23,226,508	18,230,126
Stroke incidence (2016–2030)					
Male	3,237,421	7,371,258	14,811,925	22,241,630	25,126,874
Female	3,807,198	6,885,464	11,759,056	12,473,629	10,607,893
Total	7,044,619	14,256,722	26,570,981	34,715,259	35,734,767
CVD deaths (2016–2030)					
Male	708,068	1,848,423	4,009,758	8,820,599	7,661,065
Female	205,738	651,001	2,127,829	6,476,425	6,573,086
Total	913,806	2,499,424	6,137,587	15,297,024	14,234,151

Data are based on authors’ calculations. Age strata lower limits are inclusive

*AMI* acute myocardial infarction, *CVD* cardiovascular disease

### Estimates of impact from optimal use of medication

**Fig. 2:**
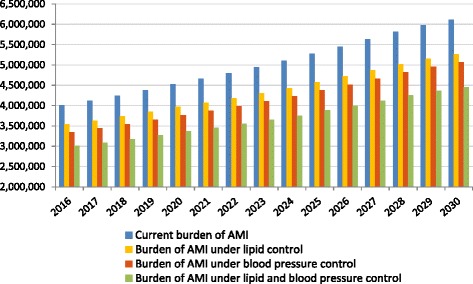
Estimates of total number of AMIs under different treatment policy scenarios (2016–2030). Notes: Data are based on authors’ calculations. AMI = acute myocardial infarction

**Fig. 3:**
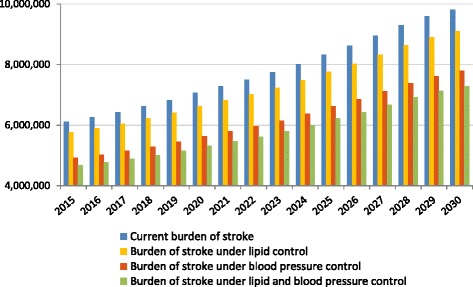
Estimates of total number of strokes under different treatment policy scenarios (2016–2030). Notes: Data are based on authors’ calculations

**Fig. 4:**
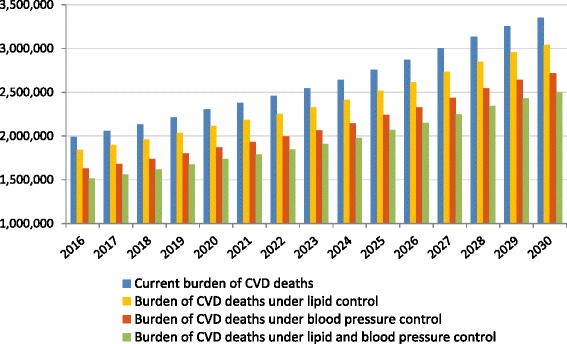
Estimates of total number of CVD deaths under different treatment policy scenarios (2016–2030). Notes: Data are based on authors’ calculations

When we applied the LDL-C shifting model, under the assumption that all people who could benefit from lipid lowering therapy do so, we predicted that the potential number of AMIs that could be avoided would be between 450,000 and 850,000 per year between 2016 and 2030. In addition, between 350,000 and 700,000 strokes and between 150,000 and 300,000 CVD deaths could be avoided per year in 2016–2030. Similarly, when we modeled the impact of optimal utilization of blood pressure medication in the Chinese population, we predicted that between 600,000 and 1 million AMIs could be avoided per year, as well as between 1.2 and 2 million strokes and between 350,000 and 600,000 deaths from CVD could be averted in the 2016–2030 period. When we modeled the impact of optimal coverage of both blood pressure and lipid control medication, the resulting events averted were estimated between 1 and 1.65 million for AMIs, 1.4 to 2.5 million for strokes, and 450,000 to 850,000 for deaths from CVD that could be prevented per year in 2016–2030. The impact on overall burden of AMIs, strokes, and CVD deaths as compared to the total burden are shown in Table 3 and Figs. 2, 3 and 4.

**Table 3:** Estimates of number of AMIs, strokes, and CVD deaths that could be prevented (2016–2030)

	AMIs averted (2016–2030)	Strokes averted (2016–2030)	CVD deaths averted (2016–2030)
*Lipid management*			
Total population	9,718,958	7,822,617	3,359,026
At least one other CVD risk factor	8,861,135	6,955,115	3,034,802
> 55 years	7,847,580	6,798,342	3,039,769
> 65 years	5,330,256	5,124,158	2,497,361
*Blood pressure management*			
Total population	12,690,319	24,090,474	7,322,172
At least one other CVD risk factor	11,705,084	21,439,156	6,570,986
> 55 years	10,850,141	21,062,444	6,822,105
> 65 years	7,150,355	14,382,099	5,453,613
*Lipid and blood pressure management*			
Total population	19,621,753	29,640,863	9,713,259
At least one other CVD risk factor	18,020,822	26,371,553	8,730,106
> 55 years	16,431,006	25,878,475	8,982,422
> 65 years	10,932,970	18,007,273	7,226,067

The three targeted approaches to lipid and blood pressure management include (i) treating all those with hyperlipidemia with a measure of LDL-C >130 mg/dL and/or hypertensives with SBP > 140 mmHg who had at least one other CVD risk factor; (ii) treating all with a measure of LDL-C >130 mg/dL (and/or SBP >140 mmHg) and ages 55 years or older; and (iii) treating all with a measure of LDL-C >130 mg/dL (and/or SBP >140 mmHg) and ages 65 years or older. Data are based on authors’ calculations

*AMI* acute myocardial infarction, *CVD* cardiovascular disease

### Social and economic value of comprehensive lipid control with statins

We estimated the social value of deaths averted, the cost of treatment net of the cost savings from events and hospitalization averted, and the net social value (social value net of net costs of treatment) for a series of scenarios. We estimated the value of treating everyone who qualifies for either lipid lowering medication, blood pressure medication, or both. We also estimated the social value for three potential targeted approaches: treating all with a measure of LDL-C >130 mg/dL and/or SBP >140 mmHg who had at least one other CVD risk factor; treating all with a measure of LDL-C >130 mg/dL and/or SBP > 140 mmHg and ages 55 years or older; and treating all with a measure of LDL-C >130 mg/dL and/or SBP > 140 mmHg and ages 65 years or older.

**Fig. 5:**
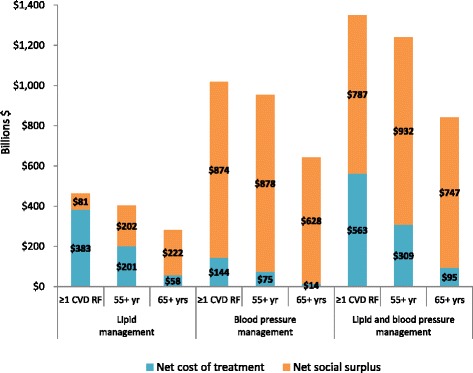
Estimates of net social value under three targeted approaches to lipid and blood pressure management. Notes: The three targeted approaches to lipid and blood pressure management include (i) treating all those with hyperlipidemia with a measure of LDL-C >130 mg/dL, and/or hypertension with SBP > 140 mmHg who had at least one other CVD risk factor; (ii) treating all with a measure of LDL-C >130 mg/dL (and/or SBP > 140 mmHg) and ages 55 years or older; and (iii) treating all with a measure of LDL-C >130 mg/dL (and/or SBP > 140 mmHg) and ages 65 years or older. Data are based on authors’ calculations. The assumed value of a life year in China is estimated at $13,408 (twice the GDP per capita in 2015). Approximately 23 % of the above estimates accrued between 2016 and 2020 and 55 % accrued between 2016 and 2025

We found that treating people with at least one other CVD risk factor with a combination of blood pressure lowering medication and lipid-lowering therapy would result in 4.3 billion person-years of treatment over the 15 year period, a net cost to the Chinese healthcare system of $563 billion and a value of life years saved of $1349 billion. The net social value, or social surplus, that results would be $787 billion, yielding a surplus to benefit ratio of 58 %. Treating only people over 55 years of age would result in 2.8 billion person-years of treatment, net cost to the Chinese healthcare system of $309 billion, and a value of life years saved of $1240 billion. This would yield a social surplus of $932 billion, yielding a surplus to benefit ratio of 75 %. Finally, treating only people over 65 years of age would generate 1.4 billion person-years of treatment, a net cost to the Chinese healthcare system of $95 billion, and a value of life years saved of $841 billion, a social surplus of $747 billion, and a surplus to benefit ratio of 89 % (Fig. 5).

## Discussion

Cardiovascular disease and other non-communicable diseases play an increasingly important role in the disease burden faced by a number of emerging market societies, including China. For example, CVD now accounts for more deaths in China than any other illness. While the transformation appears remarkable at first glance, China now finds itself on a well-worn path familiar in other developed economies. Increasingly sedentary lifestyles and dietary changes, combined with progress in the treatment of infectious disease, frequently increase the burden of CVD. Historically this transition begins with a greater prevalence of hypertensive heart disease and hemorrhagic stroke and evolves into a second stage characterized by rising rates of ischemic heart disease, diabetes, hyperlipidemia, and obesity. China is thought to be somewhere between these two stages, with CVD burden dominated by stroke over the past decade and CHD now emerging as a parallel threat.

The incidence of CVD in China is expected to continue to grow, with one study suggesting that annual CVD events will increase more than two-fold between 2010 and 2030 based on population growth alone. When factors such as projected trends in blood pressure and cholesterol are accounted for, annual CVD events are expected to grow by an additional 23 %. These estimates imply an annual increase of 21.3 million CVD events and 7.7 million CVD-related deaths by 2030 [50]. These trends are mirrored in the results of the burden estimation part of our study.

To validate our model, we compared the outputs from our baseline year against other peer-reviewed studies that look at the burden of CVD in China. Yang et al. (2013) use results from the recent *Global Burden of Disease* study and suggest CHD (or ischemic heart disease) deaths of just under one million in 2010, with various types of CVD deaths of over 3 million [1]. Both are shown to have risen rapidly since 1990 at around 3–6 % annually, which would suggest approximately 1.1 million deaths in 2013. Our model started with an estimate of CVD deaths of 1,185,064 in 2013, well within the range seen in the literature. In terms of morbidity, Moran et al. (2010) suggest that we should expect 3.2 million AMIs in 2010 and rising [50]. We estimated 3.7 million AMIs for 2013.

To determine the potential value of future policies in controlling the inevitable rise of CVD burden in China and the resulting costs to the healthcare system and the population, it is important to estimate the potential size of this burden over time and to highlight key drivers of this growth. The World Bank estimates that reducing CVD mortality by 1 % per year for 2010–2040 would save China more than $10.7 trillion, or 68 % of China’s real GDP in 2010 [51]. Our work highlights, more specifically, the potential value of intervening in just two of the CVD risk factors and predicts a net social value of up to $1.5 trillion between 2016 and 2030.

Our study has a number of limitations. We did not explicitly differentiate between primary and secondary prevention in the empirical model. Because our model relies on a regional risk data set that was taken from studies covering a population of almost 2 billion people, it may not be a perfect fit to the Chinese population and should be viewed as a meta-level model. Although major risk confounders have been stratified, there are likely to be sources of heterogenetiy that have not been included. We also focused on the potential value attributable to averted deaths, which may underestimate the social value of CVD prevention, as it excludes the considerable morbidity burden from CVD. Counter to this, although a limitation, we have excluded any adverse effects from statins and blood pressure medication since studies have suggested that the adverse effects of both sets of drugs, while noticeable, are significantly outwieghed by the benefits [52, 53]. Lastly, due to limitations on available data, many of our efficacy and risk inputs were not specific to the Chinese population. We focused on identifying parameters estimated with high levels of statistical significance, prioritized meta-analyses over individual trial results, and used the East Asia region-specific risk data from *GloboRisk*.

Two particular aspects of the social value of reducing the burden of CVD in China have been touched upon elsewhere. A recent World Bank report [51] suggested that the combination of the low reproductive rate over the past two decades and the fast aging of the population will likely place a major strain on China’s workforce in the next 20 years. Considering that, approximately half of all CVD burden is currently within the population under 65 years of age, failing to prioritize CVD control which could severely impact the quantity and quality of human capital available to China in the coming decades.

## Conclusion

Cardiovascular disease is quickly becoming the dominant cause of preventable death in emerging economies, and the speed of its future growth is a rising concern, especially in China where the impact of rapid economic growth over the past two decades is now seen in a swift health burden transition. Using evidence on the impending demographic changes to China’s population as well as empirical evidence suggesting the impact of economic growth on future prevalence of major CVD risk factors, our model predicted that the burden of CVD in China will grow rapidly in the next 15 years if left unchecked. Our study underscored this risk to the Chinese population.

In addition, we modeled the likely impact of taking an early and aggressive stance on managing hyperlipidemia along with blood pressure, the potential surplus that would accrue to the health system, and the value of intervening to society. At a time when the Chinese government is taking steps towards a more systematic approach to health care delivery, primary and secondary prevention of CVD should be high on the agenda.

### Availability of data and materials

The data supporting the conclusions of this article are included within the article, the Appendix, and 16 additional tables provided as an additional file.

## Additional files

Additional file 1: Future burden of CVD in China Appendix. (DOCX 3274 kb) Additional file 2: Future burden of CVD in China Additional Data Dictionary. (DOCX 22 kb) Additional file 3: Future burden of CVD in China Additional Data. (ACCDB 17064 kb)

## Abbreviations

AMI: acute myocardial infarction
CHD: coronary heart disease
CHNS: China Health and Nutrition Survey
CVD: cardiovascular disease
GDP: gross domestic product
LDL-C: low-density lipoprotein cholesterol
SBP: systolic blood pressure
TC: total cholesterol
WHO: World Health Organization
