# Supplementary material for: Estimating the future burden of cardiovascular disease and the value of lipid and blood pressure control therapies in China
Source: BMC Health Serv Res. 2016 May 10;16:175. doi: 10.1186/s12913-016-1420-8 (PMC4862139; doi:10.1186/s12913-016-1420-8)
Supplement: Additional file 1: — Future burden of CVD in China Appendix. (DOCX 3274 kb) [file 12913_2016_1420_MOESM1_ESM.docx]

**Estimating the future burden of cardiovascular disease and the value of lipid and blood pressure control therapies in China**

**Additional file 1 China Health and Nutrition Survey (CHNS) data**

Subjects (N=7,127) were extracted from the 2009 wave, with a complete record of data for metabolic syndrome risk factors (fasting glucose, triglyceride and high-density lipoprotein concentrations, abdominal obesity measure, systolic blood pressure), age, gender and smoking status (for males only), and low-density lipoprotein cholesterol (LDL-C) concentrations.

The metabolic syndrome cut-off values were defined as:

- Fasting glucose > 110 mg/dL
- Triglycerides > 150 mg/dL
- Reduced high-density lipoprotein < 40 mg/dL (males) and < 50 mg/dL (females)
- Waist circumference > 102 cm (males) and 88 cm (females)
- Systolic blood pressure > 130 mmHg

**2 Relative risk models**

***Incidence***

The relative risks of incidence for AMI, ischemic and hemorrhagic stroke were obtain from Moran et al. (2010) for subjects with a particular metabolic syndrome risk factor condition compared to those without the condition (Table A2.1).[5[1](#_ENREF_1)]

**Table S2.1. Relative risks for individual metabolic syndrome conditions per unit**

|  | Gender | LDL-C | Obesity (BMI) | High-density lipoprotein | Blood pressure | Diabetes |
| --- | --- | --- | --- | --- | --- | --- |
| Risk unit (U) |  | 1 mmol/L | 3% | -1 mmol/L | 20 mmHg | Yes(1)/No |
|  |  |  |  |  |  |  |
| AMI | male | 1.31 | 1.19 | 0.79 | 1.35 | 1.18 |
| AMI | female | 1.17 | 1.07 | 0.31 | 1.25 | 1.70 |
| Ischemic stroke | male | 1.12 | 1.16 | 0.68 | 1.72 | 1.64 |
| Ischemic stroke | female | 1.12 | 1.17 | 0.40 | 1.49 | 1.37 |
| Hemorrhagic stroke | male | 1 | 1 | 1 | 2.01 | 1 |
| Hemorrhagic stroke | female | 1 | 1 | 1 | 2.05 | 1 |

The relative risks were moderated by age assuming a hazard ratio per five years of:

- AMI: 1.43 (male), 1.44 (female)
- Ischemic stroke: 1.51 (male), 1.40 (female)
- Hemorrhagic stroke: 1.38 (male), 1.10 (female)

The CHNS data were used to calculate D_ik_ or the difference between the mean levels of the relevant response for condition *i* compared to those without condition *i*, for each age group *k*. The relative risks in Table A2.1 were converted to risk for a subject in age group *k* with condition *i* for event *j* (AMI, ischemic or hemorrhagic stroke), relative to a subject in age group 1 with 0 risk conditions, using the following formula:[5[1](#_ENREF_1)]

Relative risk_ijk_ = R_ij_^(D^_ik_^/U^_i_^)^ where U_i_ are the unit magnitudes in Table A2.1.

An average relative score for each subject with complete biomarker data was calculated using the above formula. These scores were then converted to relative risks for subjects with 0, 1or 2, and 3+ metabolic syndrome conditions for each age strata.

The incidence estimates given in Moran et al. (2010) were extrapolated to the base year of the model (i.e., 2012) to obtain the values shown in Table A2.2.

**Table S2.2. Incidence per 100,000 subjects in 2012**

| Event | Male | Female |
| --- | --- | --- |
| AMI | 310 | 190 |
| Ischemic stroke | 615 | 415 |
| Hemorrhagic stroke | 385 | 280 |

The relative risks derived above for risk strata 0, 1 or 2, and 3+ metabolic syndrome conditions were used to distribute the absolute values in Table A2.2, to obtain absolute incidence numbers for each risk category * age group * gender for the base year.

***Mortality***

Mortality rates for males and females with an incident AMI were obtained from Jiang et al. (2012) (shown in Table A2.3).[[6](#_ENREF_2)]

**Table S2.3. Mortality rates (per 100,000) for subjects with incident AMI**

| Age group | 35-45 | 45-55 | 55-65 | 65-75 | 75+ |
| --- | --- | --- | --- | --- | --- |
| Male mortality rate | 30.8 | 75.7 | 171.2 | 552.3 | 1925.5 |
| Female mortality rate | 7.5 | 24.8 | 90.5 | 451.2 | 1833.5 |

Mortality rates were obtained from Wang et al. (2009) for subjects who have experienced a stroke as given in Table A2.4.[[3](#_ENREF_3)]

**Table S2.4. Mortality rates by age * gender per 100,000**

| Age group | Male | Female |
| --- | --- | --- |
| 35-45 | 10.16 | 5.22 |
| 45-55 | 33.53 | 16.24 |
| 55-65 | 93.82 | 59.32 |
| 65-75 | 366.33 | 266.9 |
| 75+ | 974.7 | 763.2 |

Stroke mortality data were split into ischemic and hemorrhagic stroke groups (same for each age group) using the following ratios:

- Ratio of ischemic to hemorrhagic for males = 69.7 : 48.63
- Ratio of ischemic to hemorrhagic for females = 48.87 : 34.50

The mortality data were further divided between the metabolic syndrome risk groups using the relative risks shown in Table A2.5 (based on Malick et al. 2005).[[4](#_ENREF_4)]

**Table S2.5. Relative risk of mortality for ischemic and hemorrhagic stroke and AMI**

| Number of metabolic  syndrome conditions | AMI | Ischemic  stroke | Hemorrhagic  stroke |
| --- | --- | --- | --- |
| 0 | 1 | 1 | 1 |
| 1 or 2 | 2.1 | 1.73 | 1.73 |
| 3 or more | 3.5 | 2.71 | 2.71 |

**3 Population and epidemiological transition trend**

***Population***

The population data were extracted from the United Nations Population Division 2012 Revision (medium variant).[[31](#_ENREF_5)]

**Figure S3.1. Age stratified population trend for 2013-2030 for China**


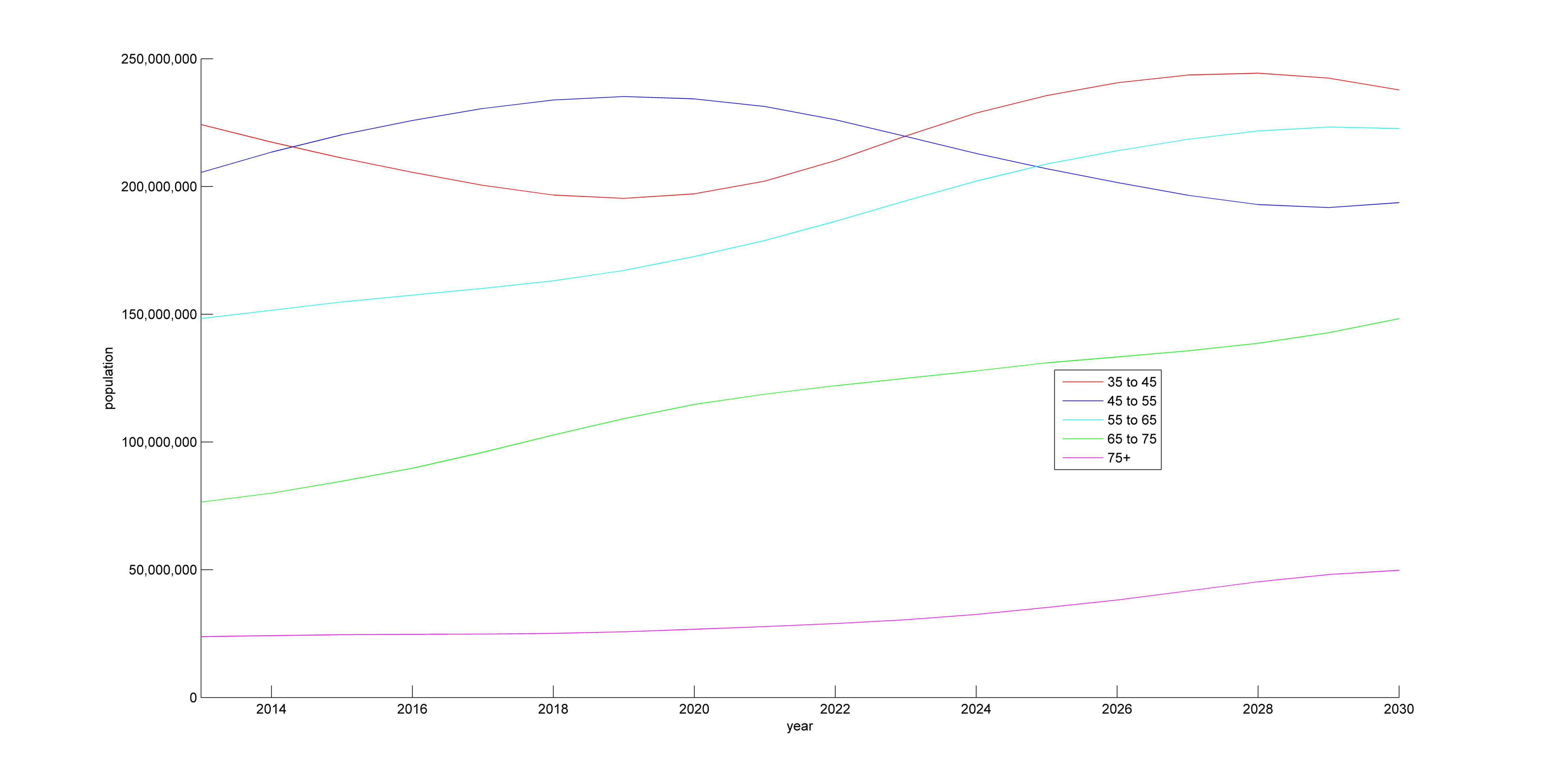


A constant 51.7% of the population was assumed to be male.

***Epidemiological transition***

Four risk factors, fasting glucose level, total cholesterol, BMI and systolic blood pressure for male and female populations were regressed against the gross national income from South East Asian countries, using a robust regression which resistant to the effect of outliers. Figures A3.2 and A3.3 show the trend lines, with the circles representing China’s gross national income (actual and projected) through 2030. The year to year percentage changes were used to predict trends in the growth of the metabolic syndrome risk factors, with total cholesterol used as a biomarker for both triglycerides and LDL-C risk factors.

**Figure S3.2. Projected epidemiological trend for males**


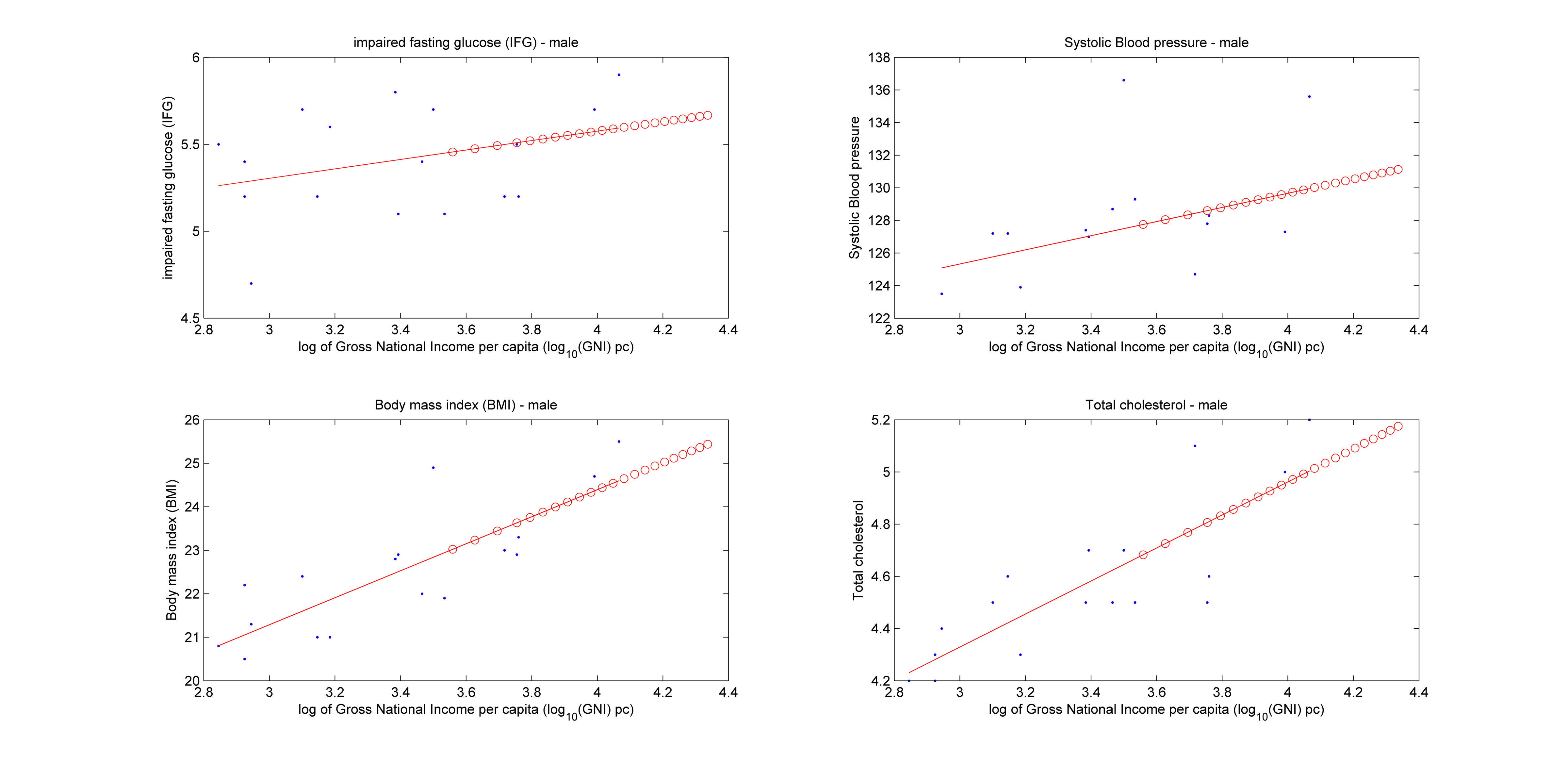


**Figure S3.3. Projected epidemiological trend for females**

**
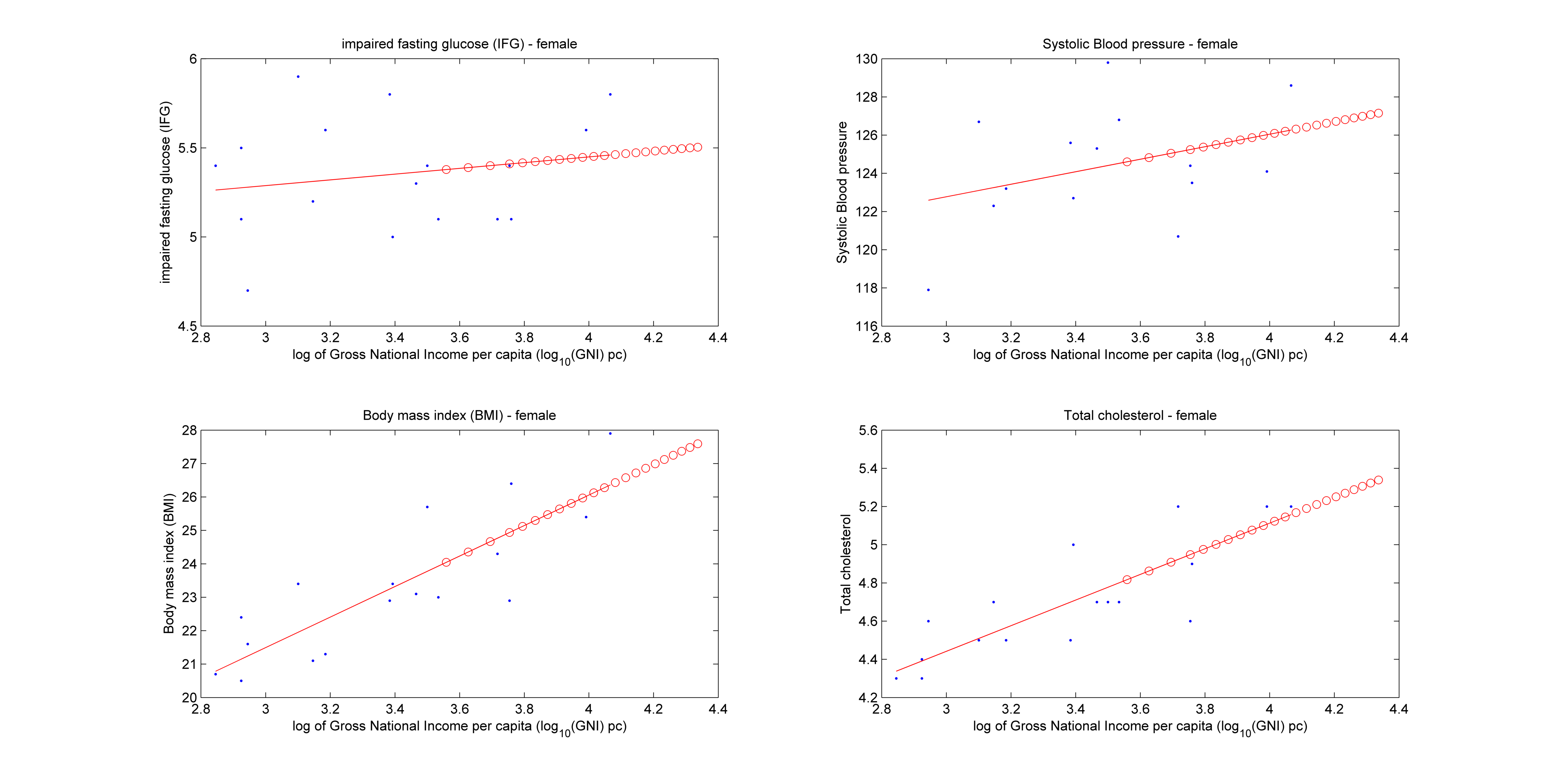
**

**4 Simulation of the effect of trend on risk factor prevalence**

For the population trend only the proportion of subjects in each age * metabolic syndrome risk category is assumed to be constant. However, for the epidemiological trend described in Appendix 3, the prevalence of the conditions underlying the risk category classification of 0, 1 or 2 and 3 to 5 changes over time.

The percentage changes per year (assumed to be the same for each age group) in the biomarker values of each condition given in Appendix 3 were applied to the baseline biomarker prevalence used to define the metabolic syndrome risk factors. This yields estimated prevalence trends over 2015-2030 for each of the five metabolic syndrome risk factors, for each gender in age group. These conditions are highly correlated and the 5*5 covariance matrices were estimated for each age group * gender group for the baseline year from the CHNS data. The covariance structure was assumed to remain constant over time.

With no closed form for the resulting trend in the prevalence in each of the condition number groups 0, 1 or 2, and 3 to 5, a simulation was used to estimate this trend. The prevalence for each risk condition and a covariance matrix can be used to construct a five variable multivariate binomial distribution. The simulation (with fixed seeds) consisted of:

1. Select strata specific prevalence and covariance matrix
2. Generate 100,000 random samples from the binomial multivariate distribution (Generating spike-trains with specified correlations)
3. Produce 100,000 rows of randomly drawn data with five columns, where each column represents yes/no for each of the five conditions
4. Calculate expected prevalence for subjects with 0, 1 or 2, 3 to 5 conditions
5. Repeat steps 1 to 4 for years 2012 to 2030
6. Repeat steps 1 to 5 for 100 different random number seeds
7. Use a smoother to estimate prevalence of condition number trend

Note: As biomarker data were only available for 2009, it is not feasible to explicitly calibrate the model using retrospective estimates.

**5 Risk aversion model**

The World Health Organization (WHO) has developed a region-specific bivariate CVD risk model [[32](#_ENREF_6)] which categorizes the risk into five levels of total cholesterol (TC) versus five levels of systolic blood pressure (SBP) for China (Western Pacific Region B model). The risk strata are further stratified into smoking/non-smoking * diabetic/non-diabetic * age * gender.

The variables TC and LDL-C are highly correlated,[[7](#_ENREF_7)] which was confirmed by the CHNS data with a highly significant (p<0.0001) relationship between TC and LDL-C (division by 38.7 converts mg/dL to mmol/L):

TC (mmol/L) = (89.50178 +0.8659873 * LDL-C (mg/dL)) / 38.7

Thus, the relationship between TC and LDL-C is used interchangeable in this risk analysis.

For each of the three risk number categories * five age category * diabetic yes/no * smoker yes/no (male only) * gender, the TC and SBP values were extracted from the CHNS data for each subject. An estimated bivariate density for SBP versus TC was then constructed from these values using a bivariate kernel density estimate with a resolution of 128 by 138. Since smoking levels for women are very low,[[8](#_ENREF_8)] smoking stratification was not modelled for females. Figure A5.1 shows the distribution of SBP versus TC values for subjects with no metabolic syndrome classes, non-diabetic, and non-smokers as the red dots with the bivariate density function estimated using a 2d kernel density estimator.

**Figure S5.1. Bivariate kernel density estimation of SBP versus TC**


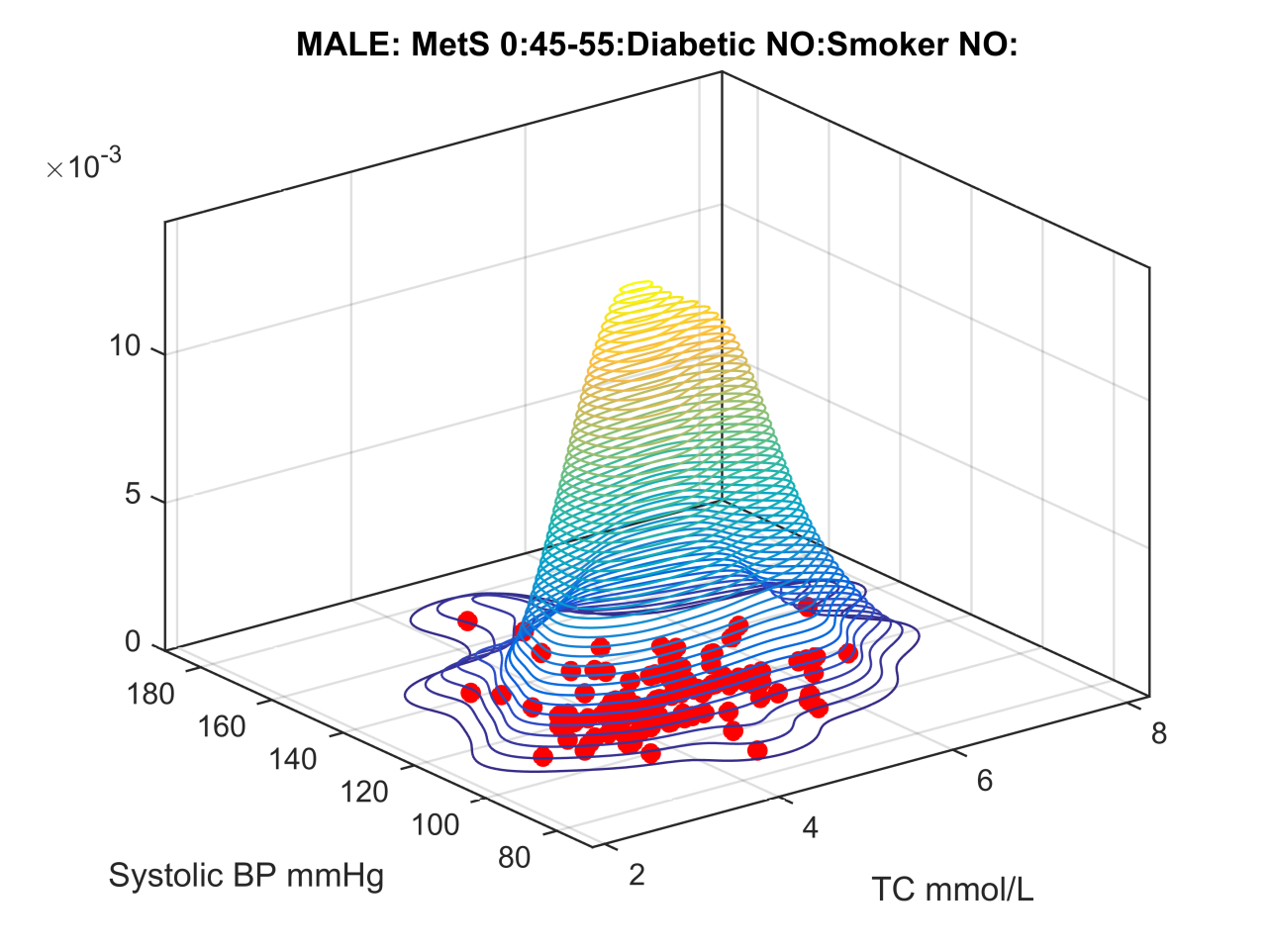


The bivariate risk distribution was combined with the bivariate density estimation to estimate the proportion of CVD events which occur across the SBP and TC values. For example, for statins assuming a prescription for subjects with LDL-C > 130mg/dL, the proportion of CVD events in these subjects was estimated as

$$P_{smoker:diabetes}\left( MetS,age \right)=\frac{\sum_{i\left( LDL-C>130 \right)} \sum_{j\left( TC \right)} A\left( i,j \right)*R\left( i,j \right)}{\sum_{i\left( LDL-C \right)} \sum_{j\left( TC \right)} A\left( i,j \right)*R\left( i,j \right)} (1)$$

where *A(i,j)* is the area of the SBP*TC cell, representing the subject population, and *R(i,j)* is the risk for that location interpolated linearly from the WHO risk model for the age/diabetes/smoking relevant risk matrix. For each metabolic syndrome * age strata four (diabetes * smoking) risk sets were estimated (two for females as smoking was ignored). These were then combined to estimate the proportion of risk in subjects with LDL-C >130 mg/dL as:

$$\sum_{diabetes} \sum_{smoking} prev\left( smoker,diabetes \right)*P_{smoker:diabetes}(MetS,age) (2)$$

where *s* and *d* refer to smoking and diabetes states (i.e., yes/no) and *prev(s, d)* is the prevalence within each smoking * diabetes combination within each metabolic syndrome * age strata, estimated from the CHNS data.

The risk proportions are then translated to risk averted by multiplying by *effect size* (e.g., 20% per 38.7 mg/dL of LDL-C) * *average reduction in LDL-C achieved by intervention.*

In the *population trend* model, we only model the risk proportion that remains constant over time and only the absolute incidence changes due to changes in population.

In the *population and epidemiological transition* model, two variables change: the percentage of diabetic subjects and the increasing trend in TC. The prevalence of diabetic subjects in equation 2 is assumed to change with this trend for each metabolic syndrome * age category from 2013 to 2030. The mean of the TC densities are assumed to change with the epidemiological transition trend. Since the density estimate is now bivariate, it is estimated as the mean of the marginal TC distribution as:

$$Mean of TC= \int x\left\{ \int f\left( x,y \right)dy \right\}dx (3)$$

where *x* and *y* represent the TC and SBP variables, and *f(x, y)* represents the bivariate kernel density estimate. The integrals are simply estimated with repeated application of the trapezium rule, and as earlier in the univariate case, the distribution is moved in the TC direction (to the right) by the amount of *% increase in mean of TC due to ET trend in year i* * *mean of TC (Met S * age) at base year*.

Equations (1) and (2) are then repeated to account for the ET trend in diabetes and TC. Although this application involves only the TC variable, SBP or both SBP and TC can be treated in a similar manner.

**6 Flow chart of modelling process**

**7 Description of calculations estimating cost of statins 2016-2030**

We took estimates of overall mean weighted price per unit for two statins, including both originator and generic form from Zeng et al. 2013.[[40](#_ENREF_9)] We combined these to estimate mean weighted cost per person per year assuming 100% adherence in the table below, and also assuming the 75% adherence seen in the trials we used to derive our efficacy data.

|  |  | **Price 2011 RMB** | **Market share** | **USD** |
| --- | --- | --- | --- | --- |
| Simvastatin | Originator drug | 3.97 | 53% | 0.64 |
|  | Generic | 2.94 | 21% | 0.47 |
| Atorvastatin | Originator drug | 9.22 | 17% | 1.48 |
|  | Generic | 7.73 | 9% | 1.24 |
|  |  |  | 100% |  |
|  | Weighted mean | 4.98 |  | 0.80 |
|  | Annual at 100% adherence | 1,819.38 |  | 291.10 |
|  | Annual at 75% adherence | 1,364.53 |  | 218.33 |

The above are 2011 prices. Zeng et al. 2014 showed that the rate at which statin prices had been falling between 2004-2013 was around 3% per year (28% over 10 years), with prices of originator drugs falling at a slightly higher rate than prices of generics.[[42](#_ENREF_10)] We applied this 3% fall to future years to produce estimates of annual patient drug costs for each year of the model.

| **Year** | **Price of 365 days (USD),**  **assuming 100% adherence** | **Price of 365 days (USD),**  **assuming 75% adherence** |
| --- | --- | --- |
| 2011 | 291.10 | 218.33 |
| 2012 | 282.37 | 211.78 |
| 2013 | 273.90 | 205.42 |
| 2014 | 265.68 | 199.26 |
| 2015 | 257.71 | 193.28 |
| 2016 | 249.98 | 187.48 |
| 2017 | 242.48 | 181.86 |
| 2018 | 235.20 | 176.40 |
| 2019 | 228.15 | 171.11 |
| 2020 | 221.30 | 165.98 |
| 2021 | 214.66 | 161.00 |
| 2022 | 208.22 | 156.17 |
| 2023 | 201.98 | 151.48 |
| 2024 | 195.92 | 146.94 |
| 2025 | 190.04 | 142.53 |
| 2026 | 184.34 | 138.25 |
| 2027 | 178.81 | 134.11 |
| 2028 | 173.45 | 130.08 |
| 2029 | 168.24 | 126.18 |
| 2030 | 163.19 | 122.40 |

**A8.1 Sensitivity analysis of social value estimates: Version of Figure 5 assuming value of a life year is 1 time GDP per capita**

**A8.2 Sensitivity analysis of social value estimates: Version of Figure 5 assuming value of a life year is 3 times GDP per capita**

**9 Life tables and methodology of estimating life years saved from premature deaths averted**

We used the most recent Global Burden of Disease [[1](#_ENREF_11)] healthy life expectancy data from China by age band and sex to estimate the life years saved from each premature death averted (Table A9.1). These data were interpolated to fit the 5 age categories used in the model to give the following estimates for health-adjusted life expectancy, shown in Figure A9.1 below.

**Figure S9.1. Interpolated health-adjusted life expectancy (HALE) by age and sex**


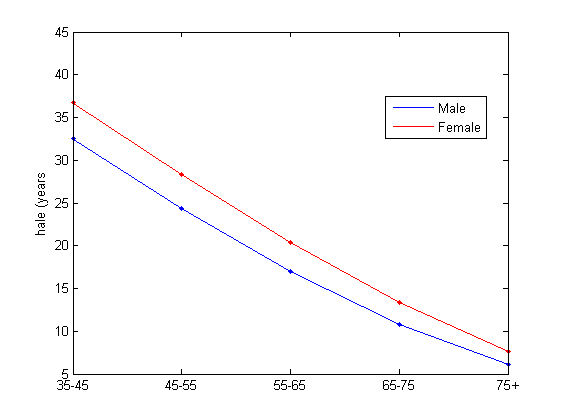


**Table S9.1. China life tables** (based on *Global Burden of Disease* *2013*)

| Age band | Sex | Health-adjusted life expectancy |
| --- | --- | --- |
| 35-39 years | Male | 34.617 |
| 35-39 years | Female | 38.898 |
| 40-44 years | Male | 30.398 |
| 40-44 years | Female | 34.561 |
| 45-49 years | Male | 26.300 |
| 45-49 years | Female | 30.327 |
| 50-54 years | Male | 22.372 |
| 50-54 years | Female | 26.228 |
| 55-59 years | Male | 18.663 |
| 55-59 years | Female | 22.284 |
| 60-64 years | Male | 15.223 |
| 60-64 years | Female | 18.506 |
| 65-69 years | Male | 12.123 |
| 65-69 years | Female | 14.959 |
| 70-74 years | Male | 9.402 |
| 70-74 years | Female | 11.705 |
| 75-79 years | Male | 7.091 |
| 75-79 years | Female | 8.826 |
| 80+ years | Male | 5.191 |
| 80+ years | Female | 6.388 |

**References**

3. Wang X, Jiang G, Choi B, Wang D, Wu T, Pan Y, et al. Surveillance of trend and distribution of stroke mortality by subtype, age, gender, and geographic areas in Tianjin, China, 1999–2006. Int J Stroke. 2009;4(3):169-74.

4. Malik S, Wong ND, Franklin SS, Kamath TV, Gilbert J, Pio JR, et al. Impact of the metabolic syndrome on mortality from coronary heart disease, cardiovascular disease, and all causes in United States adults. Circulation. 2004;110(10):1245-50.

7. Lam C, MUNRO C, Siu B. A Study of the Correlation Between Serum Total Cholesterol and Lowdensity Lipoproteins (LDL) in Chinese. Fam Pract. 1990;7(4):301-6.

8. Ng M, Freeman MK, Fleming TD, Robinson M, Dwyer-Lindgren L, Thomson B, et al. Smoking prevalence and cigarette consumption in 187 countries, 1980-2012. JAMA. 2014;311(2):183-92.
