# Supplementary material for: Estimating the future burden of cardiovascular disease and the value of lipid and blood pressure control therapies in China
Source: BMC Health Serv Res. 2016 May 10;16:175. doi: 10.1186/s12913-016-1420-8 (PMC4862139; doi:10.1186/s12913-016-1420-8)
Supplement: Additional file 2: — Future burden of CVD in China Additional Data Dictionary. (DOCX 22 kb) [file 12913_2016_1420_MOESM2_ESM.docx]

**Estimating the future burden of cardiovascular disease and the value of lipid and blood pressure control therapies in China**

The data required to estimate morbidity and mortality are contained in Appendix 2.

The Microsoft Access (2007-2013) database *Future burden of CVD in China Data.accdb* contains 16 tables of additional data described briefly below.

China_pop_Tbl The WHO population data described in Appendix 3.

costs_Tbl AMI and stroke treatment costs.

density_val_female_Tbl Kernel density estimates of systolic BP versus TC. Each bivariate kernel density estimate is a 128*128 matrix. Assuming order of counters diabetes are 1(no) to 2(yes), met S are 1 to 3 (met S risk groups), ages are 1 to 5.

The relevant matrix for diabetic*met S*age group is contained in rows 128*15*(diab-1) + 128*[(age-1) + 5*(mets-1)] + 1 to 128*15*(diab-1) + 128*[age+5*(mets-1)] (see Appendix 5).

density_pos_female_Tbl These are the x and y positions (in 2 cols) of the kernel density estimates for females (see Appendix 5).

density_val_male_Tbl As above except for males, but with a smoking category. Assume order of counters diabetes are 1(no) to 2(yes), smoking 1(no) to 2 (yes) met S are 1 to 3, ages are 1 to 5. The relevant matrix for diabetic*smoking*met S*age group is contained in rows 2*128*15*(diab-1) + 128*15*(smoke-1) + 128*[(age-1) + 5*(mets-1)] + 1 to 2*128*15*(diab-1) + 128*15*(smoke-1) + 128*[age + 5*(mets-1)] (see Appendix 5).

density_pos_male_Tbl These are the x and y positions (in 2 cols) of the kernel density estimates (see Appendix 5).

ET_trend_Tbl Simulated mean changes in met S risk groups (see Appendix 4).

ET_change_conditions_Tbl Percentage changes from baseline year of 2013 in ET trends, for diabetics, BP, TC for males and females (see Appendix 3).

marginal_val_female_Tbl Marginal means from integrating across the bivariate kernel density estimates using the trapezium rule.

Rows are 15*(diab-1) + [age+5*(mets-1)] (see Appendix 5).

marginal_val_male_Tbl As above except for males, with: rows 2*15*(diab-1) + 15*(smoke-1)+[age+5*(mets-1)] (see Appendix 5) .

MetS_differences_Tbl The differences between mean levels of met S conditions (see Appendix 2).

props_ET_Tbl From kernel density and CHNS estimates the number of subjects in each no/yes diabetes * no/yes smoking. Columns are n1, n2, n3, n4 which are no diab/no smoke, no diab/smoke, diab/no smoke, diab/smoke and N which is total. Divided into set of 15 rows (representing Met S risk group (3) * age (5), and repeated for 18 years 2013 to 2030. Note the first 18*15 rows are for females and the remainder for males (see Appendix 5).

props_ET_Tbl As above except for no ET trend. Only 30 rows required,15 for females and then final 15 for males (see Appendix 5).

WHO_risk_Tbl These are 6 by 7 risk matrices where each element gives the WHO CVD model relative risk score. Assuming smoke is 0 or 1, diab is 0 or 1, gender is 1 (male) to 2, and age is 1 to 5 the row keys A:B for extraction are:

A = 6*smoke + 12*diab + 24*(gender-1) + 48*(agey-1)+1

B = 6*smoke + 12*diab + 24*(gender-1) + 48*(agey-1)+6

Note the risk matrices are padded at boundaries assuming constant risk.

WHO_risk_BP_Tbl The SBP (30 and 310 represent the extremes of the padded boundaries) values across each of the 7 categories for TC (see Appendix 5).

WHO_risk_TC_Tbl The TC (0.1 and 13 represent the extremes of the padded boundaries) values across each of the 6 categories for SBP. (see Appendix 5).
